# Tumour Hypoxia-Mediated Immunosuppression: Mechanisms and Therapeutic Approaches to Improve Cancer Immunotherapy

**DOI:** 10.3390/cells10051006

**Published:** 2021-04-24

**Authors:** Zhe Fu, Alexandra M. Mowday, Jeff B. Smaill, Ian F. Hermans, Adam V. Patterson

**Affiliations:** 1 Malaghan Institute of Medical Research, Wellington 6042, New Zealand; rfu@malaghan.org.nz (Z.F.); ihermans@malaghan.org.nz (I.F.H.); 2 Maurice Wilkins Centre for Molecular Biodiscovery, Auckland, University of Auckland, Auckland 1142, New Zealand; a.mowday@auckland.ac.nz (A.M.M.); j.smaill@auckland.ac.nz (J.B.S.); 3 Auckland Cancer Society Research Centre, School of Medical Sciences, University of Auckland, Auckland 1142, New Zealand

**Keywords:** hypoxia, immune suppression, hypoxia-activated prodrug, tarloxotinib, CP-506, evofosfamide, HIF, oncolytic virus, checkpoint inhibitor, immunotherapy

## Abstract

The magnitude of the host immune response can be regulated by either stimulatory or inhibitory immune checkpoint molecules. Receptor-ligand binding between inhibitory molecules is often exploited by tumours to suppress anti-tumour immune responses. Immune checkpoint inhibitors that block these inhibitory interactions can relieve T-cells from negative regulation, and have yielded remarkable activity in the clinic. Despite this success, clinical data reveal that durable responses are limited to a minority of patients and malignancies, indicating the presence of underlying resistance mechanisms. Accumulating evidence suggests that tumour hypoxia, a pervasive feature of many solid cancers, is a critical phenomenon involved in suppressing the anti-tumour immune response generated by checkpoint inhibitors. In this review, we discuss the mechanisms associated with hypoxia-mediate immunosuppression and focus on modulating tumour hypoxia as an approach to improve immunotherapy responsiveness.

## 1. Introduction

Immune checkpoint inhibitors (ICIs) sit at the forefront of cancer immunotherapy. They are emerging as the primary treatment option for many advanced stage cancer patients as a result of their clinical success. Within the last decade, seven ICIs targeting the immune checkpoint receptors cytotoxic T-lymphocyte antigen 4 (CTLA-4) and programmed death protein 1 (PD-1), and the ligand to the checkpoint receptor PD-1 programme death protein ligand 1 (PD-L1) have been approved by the FDA for the treatment of a variety of malignancies [1]. While this remarkable progress has revolutionised the field of immuno-oncology, in reality, only a subset of cancer types currently respond to ICI treatments, with patients that gain long-term benefits remaining in the minority, reflected by objective response rates between 10 and 40% [2,3,4,5,6].

In general, immunotherapies are designed to increase the number and functionality of anti-tumour effector cells. The majority of such treatments target activating antigen-specific T-cells to selectively eliminate tumours through the recognition of unique or aberrantly expressed antigens presented as peptides by major histocompatibility complex (MHC) molecules on the tumour cell surface. ICIs such as anti-CTLA-4, anti-PD-1 and anti-PD-L1 monoclonal antibodies relieve T-cells from negative regulation governed by immune checkpoints by blocking the interactions between immune checkpoint receptors and their respective ligands on tumour cells, infiltrating myeloid cells or T-cells themselves [7]. The immune cell composition within the tumour microenvironment varies between patients (and sometimes between individual tumour lesions). Some have a “cold” immune privileged tumour phenotype, with minimal infiltration of immune cells (e.g., reduced numbers of effector T cells, natural killer cells and antigen presenting cells) that do not respond well to ICIs, while others possess a “hot” immune-infiltrated tumour phenotype that can respond well to ICIs [8,9]. This phenomena is suggestive of underlying resistance mechanisms that limit further advances of ICIs as monotherapy [10,11]. 

Tumours with high expression of PD-L1 respond better to ICIs targeting the PD-1/PD-L1 pathway and are also found to carry more tumour somatic mutations [12,13]. This suggests that the extent of mutagenesis in cancer cells correlates with the degree of immunogenicity of a given tumour type. A higher frequency of mutations in cellular DNA creates a greater chance of these cells at generating neoantigens which will be recognised as foreign and targeted by antigen presenting cells (APCs). Patients bearing tumours with a high mutational load are more responsive to ICIs due to increased tumour immunogenicity. For example, neoantigen signature in tumours correlated well with overall survival in patients undergoing anti-CTLA-4 treatment [12,14]. However, any increased immunogenicity can be offset by upregulating tumour expression of PD-L1 to evade immune destruction. Therefore, tumours often cannot be eradicated by the immune response.

Tumour resistance to ICIs can be classified into three broad categories: (i) primary resistance, in which an anti-tumour immune response cannot be elicited in the patient; (ii) adaptive immune resistance, in which an active anti-tumour immune response is present and can recognise cancerous cells but is unable to eliminate them due to immune evasion mechanisms established within the tumour milieu; and (iii) acquired resistance, in which the tumour initially responds to immunotherapy but subsequently becomes resistant and progresses on therapy [10,11,15]. There are numerous intrinsic and extrinsic factors that contribute to resistance to ICI treatment in patients, such as mutational load within the tumour and thus availability of tumour-associated antigens, central and peripheral tolerance mechanisms that limit the T-cell repertoire to tumour antigens, immunologically ignored tumour antigens, tumour microenvironment-associated factors (e.g., level and character of immune infiltrate, and immunosuppressive features such as tumour hypoxia), environmental factors (e.g., diet and microbiota that can alter capacity for immunity), endocrine and metabolic factors (e.g., stress response, obesity and individual variability in pharmacokinetics of treatment agents), and demographical factors (e.g., impact of sex and age on immunity) [10,11,16,17]. It is necessary for the immune response to overcome these immunosuppressive mechanisms mediated by the tumour and its microenvironment to elicit robust and durable anti-cancer immunity. In this review, we primarily focus on how tumour hypoxia, a factor within the tumour microenvironment (TME), suppresses the anti-tumour immune responses and discuss potential therapeutic strategies that can overcome tumour hypoxia to improve cancer immunotherapy, particularly ICIs.

## 2. Monoclonal Antibodies—Immune Checkpoint Inhibitors

Immunotherapy functions by stimulating the host immune system; an effective immunotherapy must be capable of generating a robust T-cell response that can overcome tumour and TME induced immunosuppression. Apart from co-stimulatory molecules, immune cells also express surface inhibitory receptors that are a part of their intrinsic regulatory mechanism, sanctioned to act as checkpoints that prevent over-activation of the immune response [7]. Such over-activation of pro-inflammatory cells with excessive production of pro-inflammatory cytokines can drive autoimmunity and unwanted damage to normal tissues. On the other hand, continuous activation of immunosuppressive cells can lead to the inhibition of T-cell activation and effector T-cell function, which dampens down the adaptive immune response against pathogens and tumours [7,18]. Not surprisingly, the immunosuppressive TME can cause upregulation of these inhibitory receptors to attenuate effector T-cell activation and function as a mechanism of tumour evasion [19]. These inhibitory receptors can be targeted with the use of monoclonal antibodies, so called ICIs, which alleviate tumour-induced immunosuppression and overcome tumour evasion by disrupting these inhibitory receptor–ligand interactions, promoting T-cell function and tumour elimination. The main advantages of ICIs over other immunotherapies is that they do not rely on tumour specific antigens and no ex vivo manipulations are required. Numerous ICIs have been developed to target inhibitory checkpoint proteins such as CTLA-4, PD-1, PD-L1, T-cell immunoglobulin mucin (TIM)-3, lymphocyte activation gene (LAG)-3, T-cell immunoglobulin and ITIM domain (TIGIT) and V-domain Ig suppressor of T cell activation (VISTA) [20]. Checkpoint proteins such as CTLA-4, PD-1, LAG-3, TIM-3, VISTA and TIGIT are mostly found on the surface of T-cells, with some also found on dendritic cells (DCs) and monocyte/macrophages (PD-L1, TIM-3, LAG-3, VISTA) or tumour cells (PD-L1, TIM-3) [18,21,22,23].

### 2.1. Approved Checkpoint Inhibitors

Ipilimumab (Yervoy™) is a monoclonal antibody that targets the CTLA-4 inhibitory receptor. It was the first checkpoint inhibitor to be approved by the FDA in 2011 for the treatment of patients with unresectable or advanced stage metastatic melanoma [24]. CTLA-4 is an essential negative regulatory component of the adaptive immune system as it maintains immunologic homeostasis by inhibiting the proliferation and activation of T-cells to suppress detrimental tissue injury due to T-cell over-activation. CTLA-4 modulates the immune response through regulating T-cell priming and T-cell activation at the early stages of an adaptive immune response by competing with CD28 for binding with the co-stimulatory molecules CD80 and CD86 on DCs to suppress T-cell activity [7,18,25]. The binding affinity of CTLA-4 is stronger than that of CD28 to CD80/86, thereby ensuring competitive inhibition of T-cell activation and proliferation by CTLA-4. CTLA-4 engagement can also induce apoptosis of activated T-cells [25]. Currently, ipilimumab is being evaluated in clinical trials for efficacy in other solid and hematologic cancers, including prostate cancer, non-small cell lung cancer (NSCLC), pancreatic cancer, ovarian cancer, glioblastoma and gastric cancer. Potential combination strategies are also being explored [18,26].

Since the FDA approval of ipilimumab, six more ICIs targeting the PD-1/PD-L1 inhibitory pathway have been approved for clinical use (Table S1). Nivolumab (Opdivo™), pembrolizumab (Keytruda™) and cemiplimab (Libtayo™) are inhibitors that target the checkpoint receptor PD-1. Anti-PD1 treatments are considered to be more favourable than anti-CTLA-4 treatments in certain tumour types due to their better safety and tolerability profiles, as well as having potential biomarkers for patient stratification and predictions for clinical outcome [6,13]. PD-1 is an inhibitory molecule that regulates the immune response after the T-cells have become activated and migrated to the peripheral tissues. Expression is upregulated on activated T-cells and provides negative feedback to attenuate local T-cell responses in order to minimise collateral tissue damage and prevent autoimmunity by binding with its two known ligands PD-L1 or PD-L2 [27].

PD-L1 is the predominant ligand for PD-1 and is highly expressed on many solid and haematological tumours including ovarian [28], breast [29], cervical [30], melanoma [31], colon [32], glioblastoma [33] and NSCLC [34] as well as DCs and macrophages [35]. Interaction between PD-1 and PD-L1 reduces cytokine production, T-cell function and proliferation, and induces T-cell apoptosis, essentially creating a negative feedback loop that dampens anti-tumour immunity. PD-L1 can also bind to CD80 which prevents its binding with CD28 on T-cells, essentially blocking CD80-mediated T-cell activation [13,18]. Tumours can evade the immune response by upregulating their PD-L1 expression to inhibit the function of tumour antigen-specific CD8^+^ T-cells. The TME can also lead to the upregulation of PD-1 on T-cells themselves, which further impairs the anti-tumour immune response [36]. The engagement of PD-1 with PD-L1 renders tumour cells refractory to apoptosis signals delivered by Fas ligation [37]. PD-L1 expression is often related to poor patient prognosis. Nevertheless, patients with higher tumour PD-L1 expression may benefit more from anti-PD-1 treatment. In clinical study, NSCLC patients whose tumoural PD-L1 expression exceeded 50% had a higher response rate to anti-PD-1, prolonged progression free survival and overall survival than those with low PD-L1 expressing tumours [13,38].

As the expression of PD-L1 is often upregulated in the TME, anti-PD-L1 antibodies that block PD-L1 have been developed as an additional strategy to target the PD-1/PD-L1 inhibitory pathway (Table S1). The FDA-approved anti-PD-L1 monoclonal antibodies are atezolizumab (Tecentriq™), avelumab (Bavencio™) and durvalumab (Imfinizi™), which prevent the interaction between PD-1 and PD-L1, and inhibit the interaction between PD-L1 and CD80 which is not targeted with anti-PD-1 antibodies [7]. Anti-PD-L1-based regimens may be more beneficial than anti-PD-1 in some disease settings. For example, studies have shown that anti-PD-L1 antibodies are associated with significantly lower rates of immune adverse events such as pneumonitis compared to anti-PD-1 in NSCLC patients across clinical trials. It is hypothesised that the reduced toxicity is due to the selective activity against PD-1/PD-L1 signalling as PD-1/PD-L2 signalling is also attenuated by anti-PD-1, which increases treatment-induced autoimmunity [39,40]. The anti-PD-L1 inhibitors also exhibit higher blocking efficiency than anti-PD-1 antibodies, with much lower half maximal effective concentrations for PD-1 signalling inhibition, meaning that a lower dose of anti-PD-L1 antibody is sufficient to induce a robust response [41]. 

### 2.2. Combination Strategies

There are currently over 2000 active ICI clinical trials (Clinicaltrials.gov, accessed 31 March 2021) with many studies designed to investigate different combination strategies involving multiple ICIs or with other treatment modalities, in hope of improving patient response rate and identifying curative combinations of agents (examples of which are illustrated in Table 1). However, despite multiple clinical trials being undertaken and evidence of improved activity with combination therapy, the proportion of responding patients still remains in the minority. This signifies the presence of resistance mechanisms in the TME which limit the development of long-term immunity against tumour antigens. Consequently, a deeper understanding of the underlying mechanisms responsible for resistance is required, including the prevalence of pathophysiological hypoxia (oxygen deprivation). 

## 3. Tumour Hypoxia and Hypoxia-Mediated Immunosuppression

### 3.1. Tumour Hypoxia

**Figure 1:**
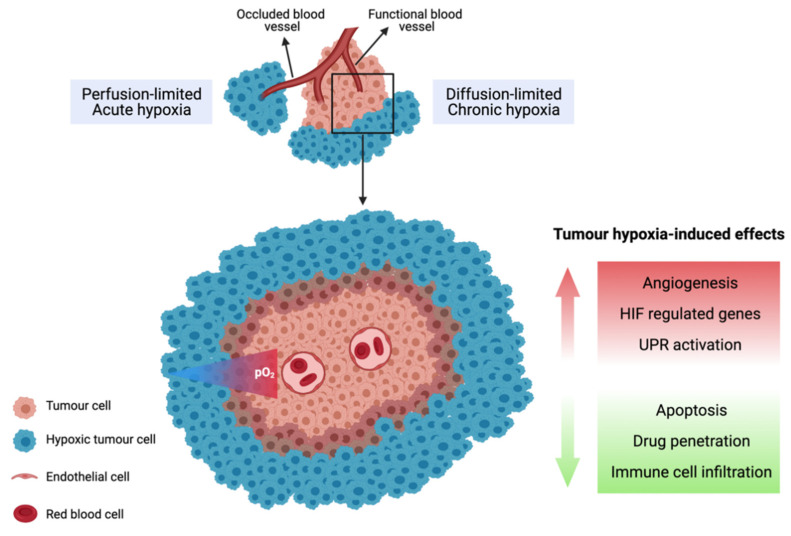
Illustration of chronic and acute tumour hypoxia. Diffusion-limited hypoxia occurs when cells are located near the diffusion limit of oxygen. Perfusion-limited hypoxia arises due to temporary occlusion of tumour vasculature.

Tumour hypoxia, defined as a state in which the oxygen levels are less than 1% O_2_ (10,000 ppm O_2_; 10 μM O_2_), is a pervasive feature of human tumours. It arises due to the abnormal structure of the tumour vasculature, leading to a mismatch between oxygen delivery and consumption [42,43]. Unlike normal tissues, the tumour vasculature lacks an organised network and is hyper-permeable, dilated and convoluted with areas of occlusions leading to poor or fluctuating blood perfusion. The rapid rate of cellular proliferation results in tumour cells also arising at an increased distance from functional blood vessels, with tumours larger than 2 mm experiencing limited oxygen and nutrient supply as well as acidosis due to inadequate waste exchange [44,45]. Cells located near the diffusion limit of oxygen (approximately 200 µm away from the blood vessels) are therefore quiescent (not rapidly proliferating) and experience diffusion-limited chronic hypoxia (Figure 1). In contrast, transient perfusion-limited hypoxia or intermittent hypoxia is caused by temporary blockage of the tumour vasculature [46]. Hypoxic tumours upregulate their expression of vascular endothelial growth factor (VEGF) to promote angiogenesis to allow for the formation of new blood vessels from existing ones to promote tumour growth and adapt to the microenvironment with limited supply of oxygen and nutrients [42,47].

### 3.2. Biological Response to Tumour Hypoxia

The hypoxic response is primarily governed by hypoxia-inducible factor (HIF)-1 transcription factor, which is a heterodimer consisting of a labile HIF-1α subunit and a constitutive HIF-1β subunit. The HIF-1α subunit is unstable under oxygenated conditions due to its regulation by prolyl hydroxylase-domain protein 1-3 (PHD1-3), which hydroxylates the oxygen-dependent degradation domains (ODD) of HIF-1α using molecular oxygen as a substrate. The hydroxylated prolines 402 and 564 are recognised by the von Hippel-Lindau (VHL) complex to target HIF-1α for degradation by the proteasome. Under hypoxic conditions, the ODD cannot be hydroxylated by PHDs, which leads to accumulation of HIF-1α and the formation of the active transcription factor HIF-1. The active HIF-1 heterodimer binds to hypoxia-responsive elements in the promoter/enhancer regions of HIF-1 regulated genes to transcriptionally activate a wide variety of genes involved in cell metabolism, angiogenesis, glucose metabolism, apoptosis, cell survival, cell proliferation and pH regulation [48,49,50]. Another HIF family transcription factor, HIF-2α, is also involved in activating hypoxic responses and is structurally similar to HIF-1α apart from the transactivation domain. This difference confers target gene specificity of HIF-1α and HIF-2α, leading to them having both overlapping and unique target genes [51,52,53]. Unlike HIF-1α, which is the main regulator of the glycolytic pathway and expressed mainly during the acute phase of hypoxia response (<24 h), HIF-2α is mostly involved in promoting an undifferentiated phenotype of pluripotent cells and drives the chronic response of hypoxia (>24 h) [54,55].

The unfolded-protein response (UPR) is also an important adaptation to severe hypoxia (reviewed in [56,57,58]). Prolonged exposure of cells to hypoxia results in endoplasmic reticulum (ER) stress and disruptions in protein folding and trafficking as the cells attempt to survive under hypoxia [56]. The accumulation of unfolded or misfolded proteins in the ER induces higher demand of binding immunoglobin protein (BiP), which initiates the UPR by dissociating from luminal domains of proteins including protein kinase RNA-like ER kinase (PERK), inositol-requiring enzyme 1α (IRE1α) and activating transcription factor 6 (ATF6) [57]. Upon dissociation, PERK and IRE1α become activated via multimerization and autophosphorylation. Activated PERK phosphorylates the eukaryotic initiation factor eIF2, leading to the translation of activating transcription factor 4 (ATF4). ATF4 then induces genes involved in oxidative stress resistance, redox homeostasis and amino acid biosynthesis [57,59]. Activated IRE1α degrades mRNAs and performs splicing of X-box binding protein transcription factor (XBP1) mRNA to generate the transcriptionally active spliced XBP1 (XBP1s). Both ATF4 and XBP1s then induce transcriptional programs to restore ER homeostasis [60]. ATF6, on the other hand, is translocated to the Golgi apparatus where it is processed by proteases to release a cytoplasmic domain ATF6f (p50). The transcriptionally active ATF6f is released to then activate a transcriptional program to restore ER homeostasis and survival [57,61]. Hypoxia-induced UPR can support the survival of cancer cells, promote angiogenesis and promote cancer cell resistance to chemotherapy [56,62].

Many tumour types contain high fractions of hypoxia such as those of the brain, head and neck, lung, breast, prostate, pancreas and cervix [42,43,63]. The relationship between tumour hypoxia and poor prognosis is firmly established, being associated with resistance to radiotherapy, chemotherapy and immunotherapy [16,42,64,65,66,67,68]. Hypoxia can affect many aspects of the tumour biology, such as the promotion of invasiveness and metastasis [69,70], suppression of apoptosis [71], induction of tumour angiogenesis [72] and altered tumour cell metabolism [73]. Moreover, it has been recognised that hypoxia is central to the generation of an immunosuppressive TME, thereby inhibiting the anti-tumour immune response [16,52,74]. As detailed in this review, tumour hypoxia can drive the suppression of the function and proliferation of effector T-cells and exacerbate tumour escape from immune surveillance, with a network of immunosuppressive cells, growth factors and cytokines being implicated in this process [16].

### 3.3. Hypoxia Promotes Immune Tolerance through Multiple Mechanisms

Tumours can evade immune recognition and destruction by cytotoxic T-cells via numerous mechanisms, including the generation of an immunosuppressive environment and development of resistance to clearance by immune effector cells [21]. An immunosuppressive environment manifests through the recruitment of immunosuppressive cells such as regulatory T-cells (Tregs) and myeloid derived suppressor cells (MDSCs), which suppress the effector function of cytotoxic T-cells through the production of suppressive factors, including tumour growth factor (TGF)-β, IL-10, VEGF, indoleamine 2, 3-dioxygenase (IDO) and arginase. These proteins, growth factors and cytokines can also promote tumour invasiveness, angiogenesis, and proliferation, as well as preventing the full activation and maturation of APCs. Immature DCs, in the absence of co-stimulatory molecules such as CD80, CD86 and CD40, promote T-cell tolerance rather than activation [21,75,76,77,78]. Similarly, tumour cells that lack the expression of co-stimulatory molecules can also induce T-cell tolerance or anergy when T-cells engage with tumour antigens present on the cell surface [79]. Further, the recruited MDSCs along with tumour associated macrophages (TAMs) create an inflammatory TME which facilitates tumour formation, progression, angiogenesis and metastasis by inducing chronic inflammation at tumour sites [21,80]. Tumours also develop resistance to cytotoxic T-cell killing by creating a defective antigen presentation pathway through the down-regulation of MHC molecules, transporter associated with antigen processing protein (TAP), and the tumour antigen itself. Defects in the antigen presentation machinery consequently lead to impaired tumour clearance and enhanced tumour progression, as cytotoxic T-cells can no longer recognise tumour antigens and exert their cytotoxic functions. Such tumour cells lack an immunogenic epitope and are thus ignored by the immune system, leading to a selective survival advantage [21,81,82,83]. The immune system itself can also contribute to tumour immune evasion through “immunoediting”, in which the immune system selectively eliminates immunogenic tumour cells, resulting in the survival of immune-resistant cancer cell clones [19,84]. 

**Figure 2:**
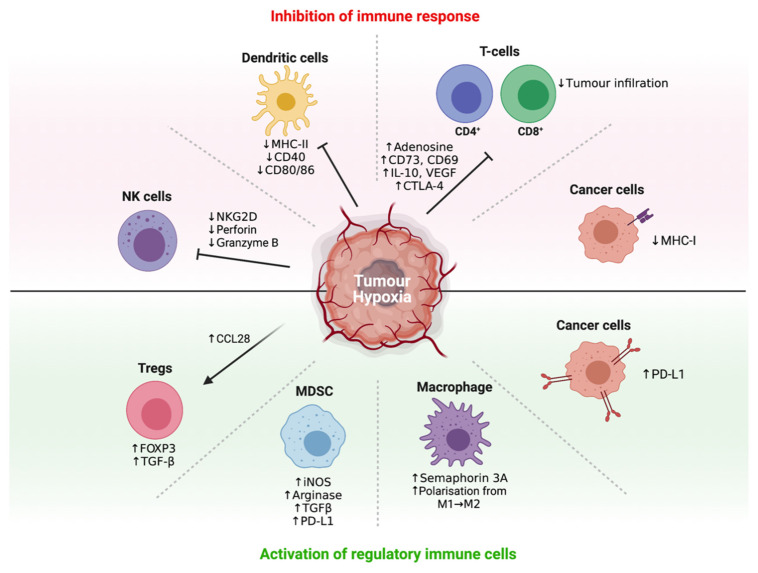
Regulation of immune response by tumour hypoxia. Different cell types by which tumour hypoxia influences in the tumour microenvironment.

Accumulating evidence indicates that most of these immunosuppressive mechanisms are orchestrated by tumour hypoxia through a network of immunosuppressive soluble factors and regulatory cell populations [16,42,43]. Specifically, tumour hypoxia has been shown to attract immunosuppressive Tregs [85], regulate the maturation and function of MDSCs [86], entrap and re-educate macrophages toward an immunosuppressive M2-like phenotype [87], and severely reduce the function of activated T-cells as a consequence of adenosine accumulation (Figure 2) [88]. Crosstalk between recruited regulatory cell populations also amplifies the production of immunosuppressive cytokines such as IL-10 and TGF-β [89]. Significantly, these effects can be reversed *directly* by hyperoxic breathing in animal models, including the restoration of activated T-cell infiltration and heightened release of pro-inflammatory cytokines and chemokines [90], indicating therapeutic interventions that suppress tumour hypoxia may hold considerable promise.

### 3.4. Hypoxia Recruits Immunosuppressive Cells to Promote Immune Tolerance

Tregs are derived from naïve CD4^+^ T-cells under the influence of TGF-β or IL-2 cytokines. Tregs are characterised by the expression of the forkhead box P3 (FOXP3) transcription factor and cell-surface molecules such as CD25, CTLA-4 and LAG-3 [19,91]. The hypoxia-induced stabilisation of HIF-1 has been shown to upregulate FOXP3, which promotes the formation of Tregs from CD4^+^ T-cells [92]. Typically, Tregs impede T-cell responses and inflammation through the secretion of immunosuppressive cytokines such as IL-10 (inhibits expression of MHC molecules and co-stimulatory molecules on APCs) and TGF-β (inhibits T-cell proliferation), or via interaction between CTLA-4 on Tregs and CD80/86 on APCs (limits T-cell priming) or by sequestering IL-2 from naïve T-cells by virtue of the high affinity receptor CD25 on Tregs. Although Tregs are essential at maintaining self-tolerance to prevent autoimmunity, their accumulation in the tumour suppresses the anti-tumour immune response [19,91]. Hypoxia also promotes the recruitment of Tregs through HIF-1-mediated induction of the C-C motif chemokine ligand (CCL)-28. CCL28 acts as a chemoattractant for Tregs through its binding to C-C motif chemokine receptor 10 (CCR10) on Tregs. The expression of CCL28 has been found to correlate with HIF-1α expression in ovarian cancer and is associated with poor patient prognosis. Similarly, hypoxia-induced recruitment of Tregs has been associated with a poor prognosis for patients with hepatocellular carcinoma (HCC) and basal-like breast cancer [85,93,94]. HIF-2α is also involved in Treg stability as HIF-2α-knockout Tregs are functionally defective at suppressing effector T-cell function. Mice with FOXP3 conditional knockout of HIF-2α also showed resistance to the growth of MC38 colon tumours and metastatic invasion of B16.F10 melanoma [95].

Hypoxia regulates the function and maturation of MDSCs, a heterogeneous group of immature immune cells of myeloid origin, consisting of immature macrophages, granulocytes and DCs. MDSCs are generated from the bone marrow and have the ability to differentiate into mature myeloid cells in the presence of the appropriate cytokines. However, their maturation is restrained under hypoxia, resulting in the accumulation of immature MDSCs in the lymphoid tissues and the tumour, leading to suppression of appropriate immune responses [74,86]. This is due to HIF-1-induced upregulation of ectonucleoside triphosphate diphosphohydrolase 2 (ENTPD2), which converts extracellular adenosine triphosphate (ATP) to 5′-adenosine monophosphate (AMP). 5′-AMP prevents the maturation of MDSCs and promotes their maintenance [96]. Tumour-associated MDSCs also upregulate the production of nitric oxide (NO) and arginase-1, leading to antigen-specific Treg proliferation as well as the suppression of antigen-specific and non-specific T-cell functions. Hypoxia/HIF-1 induces the expression of tumour-derived factors such as VEGF, GM-CSF and prostaglandins, which further contribute to the accumulation of MDSCs in the TME. HIF-1-induced upregulation of CCL26 also increases the recruitment of MDSCs that express the cognate receptor, C-X3-C motif receptor 1 (CX3CR1) [74,86,97]. HIF-1 directly regulates the expression of PD-L1, and under hypoxic conditions, PD-L1 is upregulated on MDSCs to promote T-cell anergy and tolerance [74,98]. HIF-1 also promotes the differentiation of MDSCs into immunosuppressive TAMs that further dampen down the anti-tumour immune response [86,98]. 

Macrophages are derived from myeloid progenitor cells via a monocyte precursor. Following their infiltration into solid tumours, tumour-derived cytokines such as IL-4 and IL-10 can polarise these macrophages into a so-called M2-like phenotype (F4/80^+^ CD206^+^ CD11c^-^), giving rise to TAMs that are immunosuppressive (compared to its immunostimulatory M1-like phenotype counterpart (F4/80^+^ CD206^-^ CD11c^+^)). M1-like macrophages are generally activated by IFN-γ and lipopolysaccharide (LPS), and produce high levels of IL-12 to promote the anti-tumour immune response. The abundance of M2-like macrophages in the hypoxic TME facilitates tumour progression through the production of high levels of IL-10, and by promoting angiogenesis, invasion and metastasis [99,100,101,102]. This maladaptive polarization of macrophages is intrinsically connected with the hypoxia/HIF sensors and the UPR [103]. Tumour hypoxia recruits M2-like macrophages via the HIF-1-regulated secretion of chemoattractant VEGF and endothelins, leading to their enhanced migration into the less vascularised regions of the tumour. Hypoxia-induced tumour-secreted Semaphorin 3A also contributes to M2-like macrophage recruitment to the hypoxic TME by binding to Neuropilin-1 expressed on macrophages [104]. HIF-2α is also involved in TAM accumulation in the TME and is stabilized in hypoxic macrophages. TAMs with high levels of HIF-2α correlate with increased tumour grade, and a high number of HIF-2α-expressing TAMs is associated with poor prognosis and tumour recurrence [105,106]. Further, in both murine HCC and colitis-associated cancer models, mice with HIF-2α-deficient TAMs showed reduced tumour infiltration of TAMs [107]. TAMs resident in the hypoxic areas of the tumour upregulate their expression of matrix metalloproteinase-7 protein, which cleaves Fas ligand from neighbouring cells rendering tumours less responsive to lysis by T-cells and natural killer (NK) cells [74,108]. TAMs express inducible nitric oxide synthase (iNOS) which produces NO and arginase-I, both of which suppress T-cell signal transduction and T-cell function and deplete the supply of L-arginine important for T-cell proliferation and survival. The production of iNOS and arginase-I is increased under hypoxia as their expression is mediated by HIF-1 at the transcriptional level, resulting in enhanced suppression of the anti-tumour immune response [109,110]. Not surprisingly, elevated numbers of TAMs are often associated with poor prognosis [104].

### 3.5. Hypoxia Interferes with and Suppresses Effector T-Cell, DC and NK Cell Function

Effector T-cell activity is disfavoured within the hypoxic tumour regions since HIF-1 acts as a negative regulator of effector T-cell activation and function. For example, hypoxia has been shown to reduce the expression of T-cell activation markers CD69 and CD40L [111] and studies have implicated HIF-1 in this process, as gene knockout οf HIF-1 in T-cells is sufficient to restore their proliferative phenotype and secretion of pro-inflammatory cytokines, e.g., IFN-γ [104,112]. In vitro assays have shown that T-cells cultured under 1–5% oxygen had a significant reduction in T-cell proliferative activity compared to T-cells cultured in more oxygenated conditions (21% oxygen). T-cells cultured in a lower oxygen environment also exhibited decreased IL-2 and IFN-γ production [111]. However, the precise oxygen concentration dependence of these effects is not well defined. The mechanisms by which HIF-1 suppresses the function of effector T-cells are complex, but include the upregulation of co-inhibitory receptors (e.g., CTLA-4 and LAG-3) [104,113], the differentiation of CD4^+^ T-cells into Tregs and the indirect effect of altered tumour cell metabolism [85,92]. As discussed earlier, hypoxia/HIF-1 transforms CD4^+^ T-cells into Tregs in a TGF-β-dependent manner [85,92]. The recruitment of Tregs into tumour sites suppresses the effector function of CD8^+^ T-cells. While a high effector T-cell/Treg ratio is favourable for the initiation of anti-tumour immune responses, the limited infiltration of CD4^+^ and CD8^+^ T-cells in hypoxic areas of the tumour leads to localised reductions in effector T-cell/Treg ratios and thus regional immunosuppression [16,67]. HIF-2α can also suppress T-cell function by upregulating PD-L1 expression on tumour cells. In patients with clear cell renal carcinoma, the expression of PD-L1 showed positive correlation with HIF-2α expression [114].

Changes in tumour cell metabolism also affect the function of effector T-cells within the hypoxic TME. Tumour cells often adapt to a hypoxic microenvironment by switching from oxidative phosphorylation to glycolysis, through HIF-1-mediated induction of various glycolytic enzymes to further elevate this process for ATP generation [115]. The elevated glycolytic activity in solid tumours leads to increased competition for nutrients between tumour and immune cells, as well as the increased production of glycolytic metabolites such as lactate, protons and carbonic acid, which promotes acidosis of the hypoxic TME [116,117,118]. The accumulation of lactic acid suppresses the proliferation and cytokine production activities of cytotoxic T-cells as well as inhibiting their cytolytic activity [119,120]. Further, the acidic TME impairs the secretion of proinflammatory cytokines by T-cells (e.g., IL-2, TNFs and IFN-γ) and upregulates CTLA-4 expression, rendering tumour infiltrating T-cells more susceptible to negative regulatory signals [121]. Thus, hypoxia-driven tumour acidosis promotes tumour progression and is a barrier to T-cell function in the TME.

The accumulation of extracellular adenosine is an important mechanism by which hypoxia can suppress T-cell activity. Dead and dying cells in the TME release ATP, which can be metabolised by ectonucleotidases CD73 and CD39 on the surface of immune cells. Critically, both ectonucleotidases are HIF-1 regulated, and their expression and activity is upregulated in hypoxic tumours, which leads to the increased production of cyclic adenosine monophosphate (cAMP) and adenosine, thereby enhancing immune suppression [104,122,123]. ATPs are first recognised and converted into AMPs by CD39, which are then converted by surface CD73 molecules into adenosines [104]. The cellular uptake of nucleosides is mediated by the human equilibrative nucleoside transporter 1 (ENT1), whose expression is reduced under hypoxic conditions, resulting in the accumulation of extracellular adenosine [124]. Adenosines can then bind to their receptors (A2AR and A2BR) on the immune cells to promote the production of intracellular cAMP, a factor that negatively regulates effector T-cell function and proliferation via diverse mechanisms. For example, cAMP can interfere with T-cell trafficking through the desensitisation of chemokine receptors and impairing the secretion of pro-inflammatory cytokines [67,125]. 

Effector T-cell infiltration into the tumour is hindered through the upregulation of VEGF to promote dysregulated angiogenesis, and via the downregulation of integrins (αLβ2) on vascular endothelium by upregulating IL-10 production [53,126]. Consequently, the abnormal, disorganised tumour neovasculature lacks the appropriate proteins for adhesion, attraction and extravasation of T-cells, leading to dysregulated trafficking of T-cells into the tumour bed. Furthermore, the enrichment of IL-10, VEGF and prostaglandin E2 under hypoxic conditions induces Fas-ligand expression on the tumour vasculature to promote T-cells apoptosis, ultimately leading to reduced T-cell accumulation within the TME [16]. Hypoxia via HIF-1 also reshapes the extracellular matrix by increasing collagen deposition and inducing stromal fibrosis, which also impede the accessibility of T-cells [53].

DC functions are also negatively influenced by hypoxia. Here, the expression of maturation and co-stimulatory molecules on DCs (e.g., CD40, CD80 and CD86) is downregulated, which negatively influences the activation of naïve T-cells [127]. The maturation and function of DCs are further affected by the hypoxia-induced upregulation of VEGF and IL-10 in the TME. VEGF inhibits the maturation of DCs, while IL-10 prevents the differentiation of monocytes into DCs and downregulates CCR7 expression, which alters the homing of DCs to the lymph nodes. Concurrently, hypoxia can upregulate the expression of PD-L1 on DCs to suppress T-cell function [16,74,104]. 

NK cell functions are also affected under hypoxic conditions. NK cells are cytotoxic lymphocytes belonging to the innate immune system that can directly lyse target tumour cells via the secretion of perforin and granzymes or via Fas/Fas-ligand-induced apoptosis [128]. Hypoxia/HIF can upregulate the expression of metalloproteinase ADAM10, which is responsible for the shedding of the ligand MHC-I polypeptide-related sequence A (MICA) from the surface of tumour cells. Surface MICA is a ligand for the activating receptor natural killer group 2 member D (NKG2D) on NK cells; however, soluble MICA can downregulate the expression of NKG2D on NK cells, which contributes to tumour immune evasion [129]. The expression of other activating receptors such as NKp30, NKp44 and NKp46 involved in target-recognition and killing is also down-regulated under hypoxic conditions [130]. Acidosis also increases the number of MDSCs in the tumour to inhibit the cytotoxicity of NK cells and facilitates tumour cell invasion, and is associated with poor patient prognosis [131,132]. Finally, hypoxia appears to also impair NKT cell activity through the HIF-2α-induced downregulation of Fas ligand expression and the upregulation of A2A receptors [133].

Collectively, preclinical data overwhelmingly indicate that tumour hypoxia plays a major role in regulating the function of immune cells and promoting an immunosuppressive tumour microenvironment. Clinical studies also support the association between hypoxia and immunosuppression. For example, in a cohort of 938 HNSCC patients, tumours enriched for hypoxia-responsive genes such as *HIF1A*, *VEGF* and carbonic anhydrase IX (*CAIX*) genes were strongly associated with the lack of CD8^+^ T-cell infiltrate and immune related gene signatures [134]. In a series of breast cancer surgical specimens, HIF-1 activity predicted the expression of immunosuppressive molecules including VEGF-A, IL-10 and TGF-β, and correlated with Treg infiltration [135]. The expression of HIF-1 was also positively correlated with Tregs and TGF-β has been observed in gastric cancer patient samples [136]. Further, the HIF-1-induced upregulation of VEGF has been found to directly impede T-cell activation in the ascites of ovarian cancer patients [137], leading to immune tolerance [138]. The upregulation of several gene expression clusters associated with tumour hypoxia was found in biopsy specimens of melanoma patients resistant to anti-PD-1 treatment [139]. It is notable that castration-resistant prostate cancer, colorectal cancer, and pancreatic cancer, all of which are frequently observed to be hypoxic, are typically resistant to ICI treatments [16]. 

## 4. Therapeutic Approaches That Can Modulate Tumour Hypoxia to Improve Immunotherapy Response

### 4.1. Tumour Reoxygenation as a Proof-of-Principle

Theoretically, the most direct strategy to improve T-cell function and alleviate immune suppression is to reverse tumour hypoxia by reoxygenation [15]. Studies have demonstrated the therapeutic benefit of both direct and indirect tumour reoxygenation. A preclinical study using syngeneic tumour models of murine melanoma B16 and murine sarcoma MCA205 showed that respiratory hyperoxia (60% O_2_) could decrease intratumoural hypoxia, leading to the enhanced tumour infiltration of effector T-cells, the expression of proinflammatory cytokines, and reduced Treg suppressive activity [67,90]. Furthermore, relief from hypoxia in tumour-bearing mice housed under hyperoxia conditions led to improved tumouricidal characteristics in polymorphonuclear neutrophils, with enhanced tumour control and significantly reduced tumour burden in an autochthonous mouse model of uterine cancer [140]. In a study of B16 melanoma and MC38 colon carcinoma tumour-bearing mice, the inhibition of oxygen consumption by the systemic administration of metformin led to heightened intratumoural T-cell function and drastically improved the anti-tumour activity of anti-PD-1 that was otherwise ineffective. B16-bearing mice treated with the combination of metformin and anti-PD-1 showed complete tumour regressions. This efficacy was attributed to metformin’s activity at inhibiting mitochondrial complex 1, resulting in reduced tumour cell oxidative metabolism that alleviated tumour hypoxia [141]. The anti-malarial drug atovaquone reduces oxygen consumption rates in numerous cancer cell lines by inhibiting oxidative phosphorylation at complex III of the mitochondrial electron transport chain. Administration directly alleviates hypoxia in preclinical xenograft models [142], and clinical translation of these findings in NSCLC patients demonstrated increased tumour oxygenation that was associated with the inhibition of hypoxic gene expression [143]. These data demonstrate that the elimination of hypoxia is feasible and has the potential to improve the effectiveness of various immunotherapy strategies.

### 4.2. Hypoxia-Activated Prodrugs

**Figure 3:**
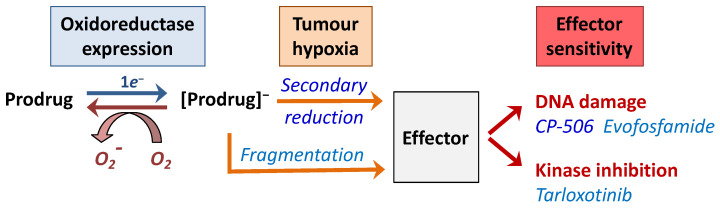
General mechanism of activation of a hypoxia-activated prodrug. Hypoxia-activated prodrugs are inactive under oxygenated conditions with their reduction limited by a futile redox cycle. Upon exposure to a hypoxic environment, HAPs undergo sequential reduction steps or fragments to form either a DNA damaging agent or release a kinase inhibitor.

In principle, relief from hypoxia-induced immunosuppression can also be achieved using hypoxia-activated prodrugs (HAPs). HAPs are agents designed to be selectively activated under hypoxic conditions to target and kill hypoxic tumour cells that are traditionally resistant to conventional therapies. HAPs are administered in an inactive form and are biologically inert in well oxygenated tissues but are capable of undergoing enzymatic reduction under hypoxic conditions to generate biologically active compounds (Figure 3) [144]. The first step of HAP activation is typically mediated by one-electron oxidoreductases which convert the inactive HAP to a prodrug radical anion that acts as a direct oxygen sensor. In normoxic tissues, the prodrug radical intermediate is rapidly back-oxidised to its original uncharged state by molecular oxygen. This futile redox cycle ensures that the prodrug remains inactive in adequately oxygenated tissues, whereas under oxygen-limiting conditions, the prodrug radical intermediate can either directly fragment or be spontaneously reduced to form an active metabolite or “effector” compound [144,145]. Consequently, appropriately designed HAPs have an improved therapeutic index compared with conventional therapy as the severe (pathological) hypoxia required for prodrug activation is not present in normal tissues. One corollary is that HAPs can generally be administered in comparatively larger doses, with the selective activation in the target hypoxic cells leading to a greater dose intensity of the released biologically active agents in the tumour [146]. 

To date, a limited series of bespoke HAPs have been evaluated in clinical trials, including porfiromycin, banoxantrone, tirapazamine, evofosfamide, PR-104 and tarloxotinib [145,147,148]. Of these, evofosfamide and tarloxotinib are currently in active clinical development, while CP-506, an analogue of PR-104, is scheduled to enter safety trials in 2021. 

#### 4.2.1. Evofosfamide

Evofosfamide (TH-302) is a 2-nitroimidazole triggered HAP of a DNA-crosslinking phosphoramidate mustard, which demonstrated considerable hypoxia-selective cytotoxicity in vitro across a range of human cancer cell lines and in vivo anti-tumour efficacy in multiple tumour xenograft models [149,150,151]. The most clinically advanced HAP thus far, evofosfamide reached Phase 3 trials in combination with gemcitabine for advanced pancreatic adenocarcinoma, before narrowly missing its primary endpoint of improvement in overall survival [152], perhaps due to a lack of appropriate patient selection [153]. The lack of T-cell infiltration in the hypoxic zones of tumours led to the hypothesis that these zones may contribute to immunotherapy resistance, and that hypoxia ablation by evofosfamide could improve the therapeutic efficacy of ICIs [154].

Using a syngeneic murine TRAMP-C2 prostate model, it was demonstrated that the improved blood vessel density observed following evofosfamide administration correlated with a reduction in hypoxic fraction and a simultaneous increase in tumour infiltration of CD3^+^ T-cells [154]. When subsequently combined with anti-PD-1 and anti-CTLA-4, proliferation of the MDSCs that are prevalent in hypoxic regions was also significantly attenuated. Together, this improved T-cell proliferation and the cytotoxic potential of CD8^+^ T-cells in the tumour, and allowed evofosfamide, in combination with CTLA-4/PD-1 blockade, to cure most animals bearing TRAMP-C2 tumours (82% overall survival) [154]. Evofosfamide also sensitized spontaneously arising prostate tumours (TRAMP transgenic mice) to combination ICI therapy via a similar mechanism: increased CD8^+^ T-cell proliferation and a significant drop in MDSC proliferation [154]. Synergy between evofosfamide and ICI has been observed in other tumour models, including HNSCC, where combination evofosfamide and anti-CTLA-4 significantly improved survival compared to anti-CTLA-4 alone [155]. Overall, these pre-clinical studies demonstrated proof-of-principle for the combination of HAPs and ICIs, and gave rise to a Phase I clinical trial evaluating the safety and tolerability of evofosfamide in combination with ipilimumab in advanced solid tumours (pancreatic, melanoma, HNSCC and prostate cancer; NCT03098160) [156]. In immunotherapy-refractory patients (n = 21), the combination therapy produced an overall response rate of 17% and a disease control rate of 83% across four dose levels. Responding patients had evidence of peripheral T-cell expansion, with increased T-cell and antigen-presenting DC infiltration into hypoxic regions of the tumour. In general, responders also had reduced proliferation of immunosuppressive TAMs [156]. Promisingly, in late 2020, the biotechnology company ImmunoGenesis announced that a Phase 2 study supported by these data will investigate evofosfamide in combination with both anti-CTLA-4 and anti-PD-1 in patients with castration-resistant prostate cancer, pancreatic ductal adenocarcinoma, and HPV-negative HNSCC [157].

#### 4.2.2. Tarloxotinib

**Figure 4:**
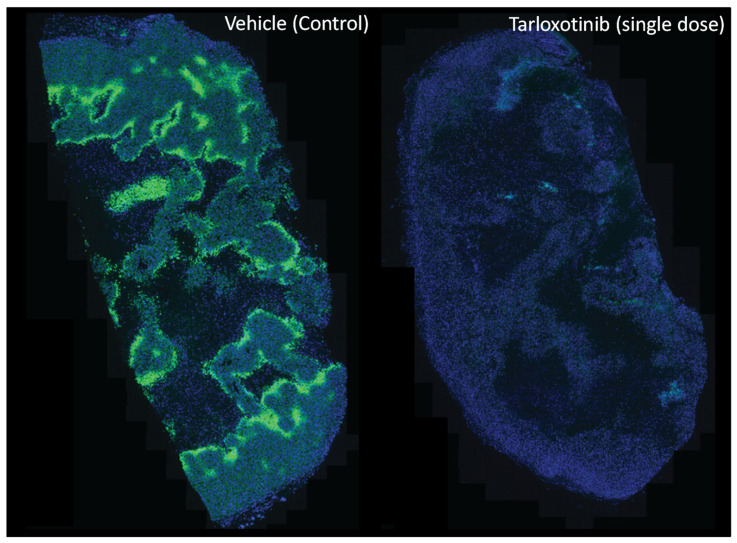
Modification of the hypoxic microenvironment following administration of tarloxotinib in PC9 mutant-EGFR NSCLC tumour-bearing mice. The representative immunohistochemical image shows hypoxic regions (green, pimonidazole binding) across a tumour cross section before and after (72 h) a single dose of tarloxotinib (30 mg/kg, intraperitoneal). Nuclei are counterstained (blue, DAPI). Image was provided by Dr Shevan Silva with permission.

Tarloxotinib is a molecularly targeted HAP developed from the prototype tyrosine kinase inhibitor prodrug SN29966 [158,159]. Tarloxotinib demonstrates strict oxygen sensitivity, with cellular metabolism completely inhibited by solution oxygen concentrations above 1 µM (0.1% O_2_ in gas phase). Under hypoxic conditions, tarloxotinib undergoes controlled fragmentation, leading to the release of a potent, irreversible, pan-human epidermal growth factor receptor (pan-HER) inhibitor (tarloxotinib-TKI) [160]. The released inhibitor, tarloxotinib-TKI, can form a covalent bond with the conserved cysteine residue within the ATP binding pocket of epidermal growth factor receptor (EGFR) (Cys797), HER2 (Cys805), and HER4 (Cys803) kinases to inhibit their downstream signalling via ERK and AKT, which ultimately leads to the inhibition of cell proliferation and survival. As tarloxotinib releases a pan-HER irreversible inhibitor, the expression of HER1-4 in tumour cells is required for target-specific cytotoxicity. For example, the administration of tarloxotinib in mice bearing the EGFR-mutant positive NSCLC tumour PC9 is observed to eliminate tumour hypoxia, as detected by pimonidazole (hypoxyprobe) binding three days later (Figure 4). 

Preclinical studies have shown the superior anti-tumour activity of tarloxotinib compared to various clinical-stage EGFR-TKIs. Notably, tumour regressions were more profound and durable compared to afatinib (irreversible EGFR inhibitor) and cetuximab (anti-EGFR monoclonal antibody) in murine xenograft models of epidermoid carcinoma (A431) and HNSCC (FaDu) [161]. Through studies using human cancer cell line derived xenografts, the remarkable anti-tumour activity of tarloxotinib was also established in NSCLC and squamous cell carcinoma of the skin, and head and neck with nanomolar potency against both the wild-type and mutant EGFR [161,162]. These favourable properties have encouraged the clinical evaluation of tarloxotinib in exon 20 insertion (Exon 20ins) EGFR mutant-positive and HER2-activating mutation NSCLC (NCT03805841) with evidence of clinical activity and low rates of EGFR-related toxicities [160,163].

Tarloxotinib has been demonstrated to enhance the efficacy of ICI. In vivo studies showed that treatment with tarloxotinib reduced the hypoxic fractions within syngeneic murine tumours and delayed tumour growth when combined with ICIs [164]. Investigation of the immune cell populations in different tissues demonstrated favourable changes in the percentage and number of different T-cell subsets and suppressor cells, leading to improvements in tumour CD8^+^ T-cell/MDSC and CD8^+^ T-cell/Treg ratios after the administration of tarloxotinib. Further studies showed increased antigen-release to the lymph nodes, increased T-cell function in the tumour and changes in cytokine production profiles after tarloxotinib administration [164,165]. Overall, tarloxotinib presents a promising HAP candidate with marked hypoxia-selectivity and anti-tumour activity to combine with immunotherapies. The use of a molecularly targeted payload (rather than a genotoxic DNA-damaging species) may avoid undesirable myelosuppressive effects that theoretically could limit the efficacy of cytotoxic HAP with ICI. 

#### 4.2.3. CP-506

CP-506 is a second generation analogue of PR-104 that is resistant to aerobic activation by aldo-keto reductase 1C3 [166,167]. CP-506 is a nitrogen mustard containing prodrug that is inactive under normoxic conditions [168]. Its oxygen dependency is attributed to the presence of the nitro group on the aromatic ring, which acts as a strong electron-withdrawing group that attracts electron density away from the non-bonding electrons of the nitrogen mustard, thus suppressing the nitrogen mustard’s ability to cross link with DNA [169,170]. Inside the hypoxic zones of the tumour, CP-506 can become metabolised by one-electron oxidoreductases to form a prodrug radical anion, which then undergoes further reduction to yield the active cytotoxins CP-506H (hydroxylamine) and CP-506M (amine). These active metabolites form DNA crosslinks by interacting with negatively charged guanine bases on DNA, leading to replication fork arrest and, ultimately, hypoxic tumour cell death [169,170]. This cytotoxicity is further amplified in cancer cells with mutations in genes involved in DNA repair pathways [167,171].

**Figure 5:**
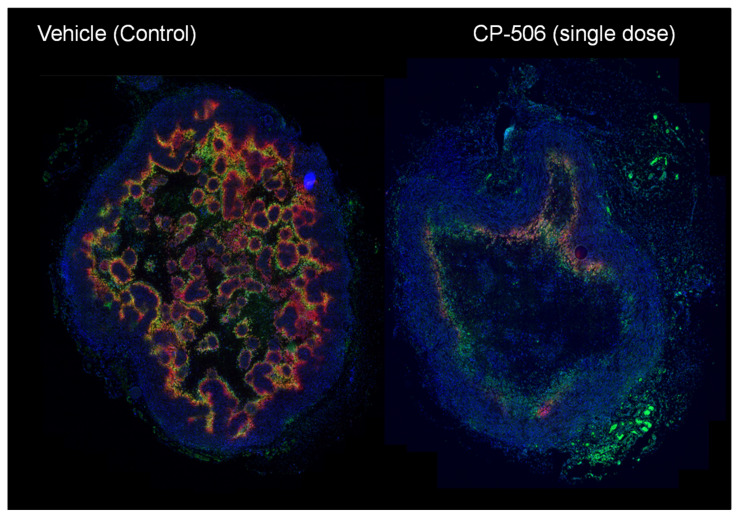
Modification of the hypoxic microenvironment following administration of CP-506 in MDA-MB-468 TNBC tumour-bearing mice. Representative immunohistochemical image showing hypoxic regions in whole tumour cross section (green, pimonidazole, red EF5 binding) before and after (24 h) a single dose of CP-506 (900 mg/kg, intraperitoneal). Nuclei are counterstained (blue, DAPI). Image was provided by Dr Maria Abbattista with permission.

CP-506 demonstrates strict oxygen sensitivity, with cellular metabolism completely inhibited by solution oxygen concentrations above 1 µM (0.1% O_2_ in gas phase). Hypoxia-selective cytotoxicity ratios up to 200-fold were observed in 2D and 3D cell culture models, and CP-506 inhibits the growth of a variety of tumour xenograft models. The observed therapeutic effect was selective for hypoxic cells and causally related to tumour oxygenation. For example, the administration of CP-506 in mice bearing the triple negative breast cancer tumour model MDA-MB-468 is observed to eliminate tumour hypoxia detected by pimonidazole and EF5 binding 24 h later (Figure 5). 

Recent evidence suggests the potential for synergy with ICI, where tumours treated with CP-506 and combination immunotherapy of L19-IL2 and anti-PD-L1 delayed the growth of tumours and prolonged median survival. In these animals, increased intratumoural immune cell infiltration was also observed [172]. 

### 4.3. Targeting of the HIF-1 Pathway

Direct inhibition of the HIF-1 pathway provides supporting evidence that removal of the hypoxia-induced immunosuppressive phenotype in the tumour microenvironment could improve the immune cell profile in the tumour, potentially boosting immunotherapy response. Knockdown of HIF-1α in murine hepatocellular carcinomas (Hep3B, HepG2 and SK-Hep-1 cell lines) led to reduced HIF-1-mediated upregulation of CCL28 expression, which can potentially lead to reduced Treg recruitment and Treg-induced tumour angiogenesis [93]. Furthermore, a study has shown that the administration of a HIF-1 inhibitor PX-478 reduced HIF-1-regulated expression of FOXP3 and VEGF. The co-administration of PX-478 with a DC-based vaccine resulted in the heightened effector function of CD8^+^ T-cells, increased T-cell proliferation and secretion of pro-inflammatory cytokine IFN-γ, in addition to reducing the function of Tregs to impede the growth of 4T1 murine breast cancer [173]. The Phase I evaluation of PX-478 was completed in 2010, but no further clinical development has been reported. 

The novel HIF-2 allosteric inhibitor belzutifan (MK-6482, PT2977) [174] is currently being evaluated in a Phase 3 trial in advanced renal cell carcinoma (NCT04195750) and VHL-associated RCC (Phase 2; NCT03401788). A dose-escalation/expansion trial in advanced solid tumours is now underway (Phase 1/2; NCT02974738). One study is evaluating belzutifan in combination with cabozantinib (NCT03634540). In addition, the HIF-2 α inhibitor PT2385 has undergone evaluation in GBM (NCT03216499) [175]. The question of whether HIF-2 inhibitors can be advantageously combined with immunotherapies remains to be determined.

Additional evidence for this hypothesis is illustrated by the targeting of the HIF-1-induced cell surface pH regulatory enzyme, carbonic anhydrase IX (CAIX). CAIX contributes to the acidification of the TME by hydrating carbon dioxide to produce protons, and its expression is associated with poor prognosis [176]. A study has shown that the ureido-sulfonamide CAIX inhibitor SLC-0111 in combination with anti-PD-1 and anti-CTLA4 increased the effector function of T-cells (granzyme B production), inhibited tumour growth, and reduced metastasis in the B16.F10 and 4T1 syngeneic tumour models [177]. Moreover, the A2AR antagonist SCH58261 that targets hypoxia-driven adenosine binding to A2AR also showed promising activity in vivo in a spontaneous HNSCC model. The administration of SCH58261 significantly delayed tumour growth by reducing the frequency of Tregs and enhancing the effector function of CD8^+^ T-cells (increased IFN-γ) [178].

### 4.4. Hypoxia Targeted Biologicals

Replicating biological vectors such as viruses or bacteria have been shown to selectively infect, replicate in, and lyse hypoxic tumour tissue, providing an alternative means to eliminate tumour hypoxia. Infection of the tumour microenvironment can often provide an immunostimulatory effect, with the potential for anti-tumour immune responses and synergy with current cancer immunotherapy strategies.

An oncolytic virus preferentially infects and lyses cancer cells, resulting in the spread of progeny virus particles to adjacent tumour cells where the oncolytic process is repeated [179]. Tropism for tumours can occur naturally (e.g., Seneca Valley virus) [180], or can be engineered through a variety of genetic modifications, including the use of tumour-specific promoters [181], modifications to viral capsid proteins to redirect virus binding [182], and dependence on signalling pathways that are constitutively activated in tumour cells [183]. Oncolytic viruses from a number of DNA and RNA families have been reported, and a small number have reached Phase III clinical testing, including Reolysin and OncoVEX GM-CSF [184]. Recent advances in the field of cancer immunotherapy have stimulated renewed interest in oncolytic virotherapy, with 62 clinical trials involving oncolytic viruses active or currently recruiting on Clinicaltrials.gov (accessed 31 March 2021).

Whilst hypoxia typically inhibits adenoviral replication [185], other types of oncolytic virus are innately more adapted to tumour hypoxia. The replication of oncolytic herpes simplex virus (HSV) is enhanced in hypoxic conditions, suggesting a natural tropism for cells with reduced oxygen tension [186]. In addition, replication of vesicular stomatitis virus (VSV) appears to be unaffected in hypoxic tumour areas [187]. Alternatively, oncolytic viruses can be genetically modified to enhance hypoxia selectivity. Hypoxia-responsive elements such as HIF-1α driven promoters can be incorporated into the viral genome to regulate the expression of essential genes for viral replication, thereby improving viral tropism for the hypoxic microenvironment [188]. However, the virus still remains dependent on the host cell translational machinery for protein synthesis, and this can be suppressed in response to hypoxic stress. Alternative approaches include “arming” viruses with oxygen-sensitive enzyme-prodrug systems to allow conditional targeted elimination of hypoxic regions [189,190].

Many genera of bacteria have been shown to specifically and preferentially target solid tumours and cause tumour cell lysis. Tumour selectivity is usually achieved by utilising the existing characteristics of the tumour microenvironment, such as necrosis and hypoxia, with these areas providing a haven for anaerobic and facultative anaerobic bacteria to germinate [191,192]. A genetically attenuated strain of the facultative anaerobe *Salmonella typhimurium* (VNP20009) is thought to target tumours due to its preference for hypoxia in tumour cores, in contrast to the more oxygenated outer regions [193]. Facultative anaerobes are able to survive in both oxygenated and hypoxic conditions, suggesting that targeted colonisation of both large solid tumours and small metastatic deposits is possible.

*Clostridium novyi*-NT spores germinate in necrotic regions of tumours and have resulted in objective responses in induced tumours in mouse models and naturally developing neoplasia in companion dogs. When injected intratumourally, *C. novyi*-NT resulted in increased phagocytosis and NK cell-like function after treatment, while intravenous injection increased LPS-induced TNF-α production, lipoteichoic acid (LTA)-induced IL-10 production and NK cell-like function post-treatment [194]. *C. novyi*-NT is currently undergoing Phase I evaluation with pembrolizumab (NCT03435952).

## 5. Concluding Remarks

Immune checkpoint inhibitors are undoubtedly a promising treatment for cancer, but their efficacy is often impeded by complex sets of tumour-intrinsic and -extrinsic resistance factors which constrain their broader therapeutic potential. The concept that tumour hypoxia within the TME is one of the major drivers for immunosuppression is becoming well established. Several studies have modulated tumour hypoxia and hypoxia-driven pathways to improve immunotherapy responsiveness with promising results. The hypoxia-targeted therapeutic strategies discussed in this review provide additional therapeutic opportunities to combine with clinical stage ICIs to increase the number of responders amongst cancer patients and improve patient outcome. More preclinical studies are needed to ensure that novel therapies that overcome hypoxia-mediated immunosuppression do so without detrimental effects on the immune system. For example, the strict oxygen-dependence of HAP activation (termed the k-curve) is an important feature given the presence of mild physiological hypoxia in various lymphoid organs [111,195,196]. The sequence and timing of the combinations will also need to be optimised. A prognostic molecular classifier based on both hypoxia status and immune response genes has been developed and validated in HNSCC [197], which may be useful to guide future clinical trial design and patient selection for therapeutic approaches incorporating both hypoxia targeting strategies and immunotherapy treatment modalities for clinical translation to improve patient prognosis.

## Figures and Tables

**Table 1:** Examples of combination immunotherapy strategies currently recruiting for clinical trial.

Combination Strategy	Examples	Phase	Clinical Trial Identifier
Chemotherapy	Pembrolizumab + usual chemotherapy for NSCLC	III	NCT04267848
	Atezolizumab + combination chemotherapy	III	NCT02912559
	Nivolumab + carboplatin and paclitaxel	III	NCT04444921
Radiotherapy	Pembrolizumab + stereotactic body radiation therapy	III	NCT03867175
	Atezolizumab + radiation therapy	II/III	NCT04402788
	Sintilimab + stereotactic body radiation therapy	II/III	NCT04167293
Multiple ICI agents	Pembrolizumab + Ipilimumab	II	NCT03873818
	Tiragolumab + Atezolizumab	I	NCT02794571
Molecular targets	Nivolumab + Lenvatinib	II	NCT03841201
	Atezolizumab + Vemurafenib and/or Cobimetinib	II	NCT04722575
	Pembrolizumab + Idelalisib	I/II	NCT03257722
Immunostimulatory agents	Nivolumab + CMP-001 (TLR-9 agonist)	II	NCT04401995
	Nivolumab (+ Ipilimumab) + OTSGC-A24 (peptide vaccine)	I	NCT03784040
	Tislelizumab + BGB-A445 (anti-OX40 agonist)	I	NCT04215978
Replicating biological vectors	Nivolumab + Talimogene laherparepvec Pembrolizumab + *Clostridium novyi*	III	NCT04330430NCT03435952

## Data Availability

Not Applicable.

## Supplementary Materials

The following are available online at https://www.mdpi.com/article/10.3390/cells10051006/s1, Table S1: Examples of checkpoint inhibitors approved by the FDA for clinical use as a monotherapy.
