# Supplementary material for: Tumour Hypoxia-Mediated Immunosuppression: Mechanisms and Therapeutic Approaches to Improve Cancer Immunotherapy
Source: Cells. 2021 Apr 24;10(5):1006. doi: 10.3390/cells10051006 (PMC8146304; doi:10.3390/cells10051006)
Supplement: Supplementary file 1 [file cells-10-01006-s001.zip › cells-1189345-SI.pdf]

Table S1. Examples of checkpoint inhibitors approved by the FDA for clinical use as a monotherapy. ORR, objective response rate; OS, overall survival; NSCLC, non-small cell lung cancer; HNSCC, head and neck squamous cell carcinoma; SCLC, small cell lung cancer; MSI, microsatellite instability; MMR, mismatch repair. HPV, human papilloma virus.

Target	Drug name	Trade name	Tumour type	Year approved	Pivotal trial	Key findings	References
CTLA-4	Ipilimumab	Yervoy	Melanoma	2011	MDX010-020	Improved the median OS by 3.7 months and 2-year survival by 9.7% compared to gp100 vaccine alone.	[1, 2]
PD-1	Nivolumab	Opdivo	Melanoma	2014	CheckMate-037	Improved ORR (31.7% vs. 10.6%) with fewer toxicities compared to dacarbazine group.	[3]
			NSCLC	2015	CheckMate-017	Improved median OS (9.2 months vs. 6 months) and ORR (20% vs. 9%) compared to docetaxel group.	[4]
			Renal cell carcinoma	2015	CheckMate-025	Improved OS (25 months vs. 19.6 months) and ORR (25% vs. 5%) compared to everolimus group.	[5]
			HNSCC	2016	CheckMate-141	Improved median OS (7.5 months vs. 5.1 months) and ORR (13.3% vs. 5.8%) compared to standard therapy.	[6]
			Urothelial carcinoma	2017	CheckMate-275	Improved objective response and OS benefit of 7 months observed, irrespective of PD-L1 expression in patients with recurrent or progressed tumours	[7]
			MSI-high, MMR-deficient colorectal cancer	2017	CheckMate-142	Patients who progressed after previous treatments showed good ORR (31.1%) and 51/74 patients had disease control > 12 weeks	[8]
			Hepatocellular carcinoma	2017	CheckMate-040	Manageable safety profile and durable objective response (ORR 20%) in patients .	[9]
			SCLC	2018	CheckMate-032	Increased overall response rate and response duration in patients after failure of platinum-based therapy	[10]
			Oesophageal squamous cell carcinoma	2020	ATTRACTION-3	Improved OS (10.9 months vs. 8.4 months) compared to chemotherapy group.	[11]
	Pembrolizumab	Keytruda	Melanoma	2014	NCT01295827	ORR of 26% in anti-CTLA-4 refractory patients.	[12]
			NSCLC	2015	KEYNOTE-010	Improved median OS (12.7 months vs. 8.5 months) and ORR (29% vs. 8%) compared to docetaxel group.	[13]
			HNSCC	2016	KEYNOTE-012	Overall ORR of 18% regardless of HPV status in patients who progressed on platinum-based therapy	[14]
			Urothelial carcinoma	2017	KEYNOTE-045	Improved median OS (10.3 months vs. 7.4 months), ORR (21.1% vs, 11.4%) and lower toxicities compared	[15]

						to chemotherapy in patients with progressed or recurred tumours after platinum-based therapy	
			MSI-high, MMR-deficient solid tumours of any histology	2017	KEYNOTE-012, -016, -028, -158 and -164	Overall response rate of approximately 40% across multiple trials involving patients with MSI-high and MMR-deficient solid tumours.	[16, 17]
			Gastric and gastroesophageal carcinoma	2017	KEYNOTE-059	ORR of 11.6% with complete response observed in 2.3% patients. Median response duration of 8.4 months in previously treated patients.	[18]
			Cervical cancer	2018	KEYNOTE-158	ORR of 12.2% with manageable safety in previously treated patients.	[19]
			Merkel cell carcinoma	2018	KEYNOTE-017	Improved ORR of 56% and 24-months overall survival rate of 68.7% in patients compared to historical chemotherapy data.	[20]
			Non-muscle invasive bladder cancer	2020	KEYNOTE-057	Complete response observed in 41% of patients with a median duration of response of 16.2 months.	[21, 22]
	Cemiplimab	Libtayo	Squamous cell carcinoma	2018	NCT02383212 NCT02760498	Response observed in 50% (phase I expansion cohorts) and 47% (phase II metastatic-disease cohorts) of patients.	[23]
			NSCLC	2021	NCT03088540	Improved OS and progression-free survival.	[24]
PD-L1	Atezolizumab	Tecentriq	Urothelial carcinoma	2016	IMvigor210	Improved OS (8.6 months) and overall ORR of 15% in patients. Patients with increased expression of PD-L1 on tumour infiltrating immune cells were more responsive to treatment (26% in PD-L1$^+$ vs. 9.5% in PD-L1$^-$ patients)	[25, 26]
			NSCLC	2016	POPLAR<br><br>OAK	Improved OS (12.6 months vs. 9.7 months) and fewer grade 3-4 adverse events (11% vs. 39%) compared to docetaxel group.<br>Improved median OS (13.8 months vs. 9.6 months) compared to docetaxel group.	[27, 28]
	Avelumab	Bavencio	Merkel cell carcinoma	2017	JAVELIN Merkel 200	ORR of 31.8% with 8 complete responses and was well-tolerated without any grade 4 events.	[29]
			Urothelial carcinoma	2017	JAVELIN Solid Tumor	ORR of 16.5%, median duration of response of 20.5 months and median OS of 7 months were observed in	[30]

						patients with progressed tumour after platinum-based therapy	
	Durvalumab	Imfinizi	Urothelial carcinoma	2017	NCT01693562	ORR of 17.8% with 7 complete response. ORR higher in PD-L1$^+$ patients (27.6% vs. 5.1%). Grade 3-4 adverse events in 6.8% patients.	[31]
			NSCLC	2018	PACIFIC	Improved median progression-free survival (17.2 months vs. 5.6 months) and ORR (30% vs. 17.8%) compared to placebo group.	[32]

References

1. Hodi FS, O'Day SJ, McDermott DF, Weber RW, Sosman JA, Haanen JB, et al. Improved survival with ipilimumab in patients with metastatic melanoma. N Engl J Med. 2010;363(8):711-23.doi: 10.1056/NEJMoa1003466.
2. McDermott D, Haanen J, Chen TT, Lorigan P, O'Day S. Efficacy and safety of ipilimumab in metastatic melanoma patients surviving more than 2 years following treatment in a phase III trial (MDX010-20). Annals of Oncology. 2013;24(10):2694-8.doi: https://doi.org/10.1093/annonc/mdt291.
3. Weber JS, D'Angelo SP, Minor D, Hodi FS, Gutzmer R, Neyns B, et al. Nivolumab versus chemotherapy in patients with advanced melanoma who progressed after anti-CTLA-4 treatment (CheckMate 037): a randomised, controlled, open-label, phase 3 trial. The Lancet Oncology. 2015;16(4):375-84.doi: https://doi.org/10.1016/S1470-2045(15)70076-8.
4. Brahmer J, Reckamp KL, Baas P, Crinò L, Eberhardt WEE, Poddubskaya E, et al. Nivolumab versus Docetaxel in Advanced Squamous-Cell Non–Small-Cell Lung Cancer. New England Journal of Medicine. 2015;373(2):123-35.doi: 10.1056/NEJMoa1504627.
5. Motzer RJ, Escudier B, McDermott DF, George S, Hammers HJ, Srinivas S, et al. Nivolumab versus Everolimus in Advanced Renal-Cell Carcinoma. N Engl J Med. 2015;373(19):1803-13.doi: 10.1056/NEJMoa1510665.
6. Ferris RL, Blumenschein G, Jr., Fayette J, Guigay J, Colevas AD, Licitra L, et al. Nivolumab for Recurrent Squamous-Cell Carcinoma of the Head and Neck. N Engl J Med. 2016;375(19):1856-67.doi: 10.1056/NEJMoa1602252.
7. Sharma P, Retz M, Siefker-Radtke A, Baron A, Necchi A, Bedke J, et al. Nivolumab in metastatic urothelial carcinoma after platinum therapy (CheckMate 275): a multicentre, single-arm, phase 2 trial. The Lancet Oncology. 2017;18(3):312-22.doi: https://doi.org/10.1016/S1470-2045(17)30065-7.
8. Overman MJ, McDermott R, Leach JL, Lonardi S, Lenz H-J, Morse MA, et al. Nivolumab in patients with metastatic DNA mismatch repair-deficient or microsatellite instability-high colorectal cancer (CheckMate 142): an open-label, multicentre, phase 2 study. The Lancet Oncology. 2017;18(9):1182-91.doi: https://doi.org/10.1016/S1470-2045(17)30422-9.
9. El-Khoueiry AB, Sangro B, Yau T, Crocenzi TS, Kudo M, Hsu C, et al. Nivolumab in patients with advanced hepatocellular carcinoma (CheckMate 040): an open-label, non-comparative, phase 1/2 dose escalation and expansion trial. The Lancet. 2017;389(10088):2492-502.doi: https://doi.org/10.1016/S0140-6736(17)31046-2.
10. Antonia SJ, López-Martin JA, Bendell J, Ott PA, Taylor M, Eder JP, et al. Nivolumab alone and nivolumab plus ipilimumab in recurrent small-cell lung cancer (CheckMate 032): a multicentre, open-label, phase 1/2 trial. The Lancet Oncology. 2016;17(7):883-95.doi: https://doi.org/10.1016/S1470-2045(16)30098-5.
11. Kato K, Cho BC, Takahashi M, Okada M, Lin CY, Chin K, et al. Nivolumab versus chemotherapy in patients with advanced oesophageal squamous cell carcinoma refractory or intolerant to previous chemotherapy (ATTRACTION-3): a multicentre, randomised, open-label, phase 3 trial. Lancet Oncol. 2019;20(11):1506-17.doi: 10.1016/s1470-2045(19)30626-6.
12. Robert C, Ribas A, Wolchok JD, Hodi FS, Hamid O, Kefford R, et al. Anti-programmed-death-receptor-1 treatment with pembrolizumab in ipilimumab-refractory advanced melanoma: a randomised dose-comparison cohort of a phase 1 trial. The Lancet. 2014;384(9948):1109-17.doi: https://doi.org/10.1016/S0140-6736(14)60958-2.

13. Herbst RS, Baas P, Kim D-W, Felip E, Pérez-Gracia JL, Han J-Y, et al. Pembrolizumab versus docetaxel for previously treated, PD-L1-positive, advanced non-small-cell lung cancer (KEYNOTE-010): a randomised controlled trial. The Lancet. 2016;387(10027):1540-50.doi: https://doi.org/10.1016/S0140-6736(15)01281-7.
14. Seiwert TY, Burtness B, Mehra R, Weiss J, Berger R, Eder JP, et al. Safety and clinical activity of pembrolizumab for treatment of recurrent or metastatic squamous cell carcinoma of the head and neck (KEYNOTE-012): an open-label, multicentre, phase 1b trial. The Lancet Oncology. 2016;17(7):956-65.doi: https://doi.org/10.1016/S1470-2045(16)30066-3.
15. Bellmunt J, de Wit R, Vaughn DJ, Fradet Y, Lee J-L, Fong L, et al. Pembrolizumab as Second-Line Therapy for Advanced Urothelial Carcinoma. New England Journal of Medicine. 2017;376(11):1015-26.doi: 10.1056/NEJMoa1613683.
16. Boyiadzis MM, Kirkwood JM, Marshall JL, Pritchard CC, Azad NS, Gulley JL. Significance and implications of FDA approval of pembrolizumab for biomarker-defined disease. J Immunother Cancer. 2018;6(1):35.doi: 10.1186/s40425-018-0342-x.
17. Diaz L, Marabelle A, Kim TW, Geva R, Van Cutsem E, André T, et al. 386P - Efficacy of pembrolizumab in phase 2 KEYNOTE-164 and KEYNOTE-158 studies of microsatellite instability high cancers. Annals of Oncology. 2017;28:v128-v9.doi: https://doi.org/10.1093/annonc/mdx367.020.
18. Fuchs CS, Doi T, Jang RW, Muro K, Satoh T, Machado M, et al. Safety and Efficacy of Pembrolizumab Monotherapy in Patients With Previously Treated Advanced Gastric and Gastroesophageal Junction Cancer: Phase 2 Clinical KEYNOTE-059 Trial. JAMA oncology. 2018;4(5):e180013-e.doi: 10.1001/jamaoncol.2018.0013.
19. Chung HC, Ros W, Delord J-P, Perets R, Italiano A, Shapira-Frommer R, et al. Efficacy and Safety of Pembrolizumab in Previously Treated Advanced Cervical Cancer: Results From the Phase II KEYNOTE-158 Study. Journal of Clinical Oncology. 2019;37(17):1470-8.doi: 10.1200/jco.18.01265.
20. Nghiem P, Bhatia S, Lipson EJ, Sharfman WH, Kudchadkar RR, Brohl AS, et al. Durable Tumor Regression and Overall Survival in Patients With Advanced Merkel Cell Carcinoma Receiving Pembrolizumab as First-Line Therapy. Journal of Clinical Oncology. 2019;37(9):693-702.doi: 10.1200/jco.18.01896.
21. Kamat AM, Shore N, Hahn N, Alanee S, Nishiyama H, Shariat S, et al. KEYNOTE-676: Phase III study of BCG and pembrolizumab for persistent/recurrent high-risk NMIBC. Future Oncol. 2020;16(10):507-16.doi: 10.2217/fon-2019-0817.
22. Balar AV, Kulkarni GS, Uchio EM, Boormans J, Mourey L, Krieger LEM, et al. Keynote 057: Phase II trial of Pembrolizumab (pembro) for patients (pts) with high-risk (HR) nonmuscle invasive bladder cancer (NMIBC) unresponsive to bacillus calmette-guérin (BCG). Journal of Clinical Oncology. 2019;37(7_suppl):350-.doi: 10.1200/JCO.2019.37.7_suppl.350.
23. Migden MR, Rischin D, Schmults CD, Guminski A, Hauschild A, Lewis KD, et al. PD-1 Blockade with Cemiplimab in Advanced Cutaneous Squamous-Cell Carcinoma. New England Journal of Medicine. 2018;379(4):341-51.doi: 10.1056/NEJMoa1805131.
24. Sezer A, Kilickap S, Gümüş M, Bondarenko I, Özgüroğlu M, Gogishvili M, et al. Cemiplimab monotherapy for first-line treatment of advanced non-small-cell lung cancer with PD-L1 of at least 50%: a multicentre, open-label, global, phase 3, randomised, controlled trial. The Lancet. 2021;397(10274):592-604.doi: 10.1016/S0140-6736(21)00228-2.
25. Necchi A, Joseph RW, Loriot Y, Hoffman-Censits J, Perez-Gracia JL, Petrylak DP, et al. Atezolizumab in platinum-treated locally advanced or metastatic urothelial carcinoma: post-progression outcomes from the phase II IMvigor210 study. Annals of Oncology. 2017;28(12):3044-50.doi: https://doi.org/10.1093/annonc/mdx518.
26. Rosenberg JE, Hoffman-Censits J, Powles T, van der Heijden MS, Balar AV, Necchi A, et al. Atezolizumab in patients with locally advanced and metastatic urothelial carcinoma who have progressed following treatment with platinum-based chemotherapy: a single-arm, multicentre, phase 2 trial. Lancet. 2016;387(10031):1909-20.doi: 10.1016/s0140-6736(16)00561-4.
27. Fehrenbacher L, Spira A, Ballinger M, Kowanetz M, Vansteenkiste J, Mazieres J, et al. Atezolizumab versus docetaxel for patients with previously treated non-small-cell lung cancer (POPLAR): a multicentre, open-label, phase 2 randomised controlled trial. The Lancet. 2016;387(10030):1837-46.doi: https://doi.org/10.1016/S0140-6736(16)00587-0.
28. von Pawel J, Bordoni R, Satouchi M, Fehrenbacher L, Cobo M, Han JY, et al. Long-term survival in patients with advanced non–small-cell lung cancer treated with atezolizumab versus docetaxel: Results from the randomised phase III OAK study. European Journal of Cancer. 2019;107:124-32.doi: https://doi.org/10.1016/j.ejca.2018.11.020.

29. Kaufman HL, Russell J, Hamid O, Bhatia S, Terheyden P, D'Angelo SP, et al. Avelumab in patients with chemotherapy-refractory metastatic Merkel cell carcinoma: a multicentre, single-group, open-label, phase 2 trial. The Lancet Oncology. 2016;17(10):1374-85.doi: https://doi.org/10.1016/S1470-2045(16)30364-3.
30. Apolo AB, Ellerton JA, Infante JR, Agrawal M, Gordon MS, Aljumaily R, et al. Avelumab as second-line therapy for metastatic, platinum-treated urothelial carcinoma in the phase Ib JAVELIN Solid Tumor study: 2-year updated efficacy and safety analysis. J Immunother Cancer. 2020;8(2):e001246.doi: 10.1136/jitc-2020-001246.
31. Powles T, O'Donnell PH, Massard C, Arkenau HT, Friedlander TW, Hoimes CJ, et al. Efficacy and Safety of Durvalumab in Locally Advanced or Metastatic Urothelial Carcinoma: Updated Results From a Phase 1/2 Open-label Study. JAMA oncology. 2017;3(9):e172411.doi: 10.1001/jamaoncol.2017.2411.
32. Antonia SJ, Villegas A, Daniel D, Vicente D, Murakami S, Hui R, et al. Overall Survival with Durvalumab after Chemoradiotherapy in Stage III NSCLC. N Engl J Med. 2018;379(24):2342-50.doi: 10.1056/NEJMoa1809697.
